# A five-year observational prospective mono-center study of the efficacy of alemtuzumab in a real-world cohort of patients with multiple sclerosis

**DOI:** 10.3389/fneur.2023.1265354

**Published:** 2023-09-21

**Authors:** Sofia Sandgren, Lenka Novakova, Anna Nordin, Markus Axelsson, Clas Malmeström, Henrik Zetterberg, Jan Lycke

**Affiliations:** ^1^Department of Clinical Neuroscience, Institute of Neuroscience and Physiology, Sahlgrenska Academy, University of Gothenburg, Gothenburg, Sweden; ^2^Region Västra Götaland, Department of Neurology, Sahlgrenska University Hospital, Gothenburg, Sweden; ^3^Laboratory for Clinical Immunology, Sahlgrenska Academy, University of Gothenburg, Gothenburg, Sweden; ^4^Department of Psychiatry and Neurochemistry, Institute of Neuroscience and Physiology, Sahlgrenska Academy, University of Gothenburg, Mölndal, Sweden; ^5^Clinical Neurochemistry Laboratory, Sahlgrenska University Hospital, Mölndal, Sweden; ^6^Department of Neurodegenerative Disease, University College London (UCL) Queen Square Institute of Neurology, London, United Kingdom; ^7^UK Dementia Research Institute at University College London (UCL), London, United Kingdom; ^8^Hong Kong Center for Neurodegenerative Diseases, Hong Kong, Hong Kong SAR, China; ^9^Wisconsin Alzheimer’s Disease Research Center, University of Wisconsin School of Medicine and Public Health, University of Wisconsin-Madison, Madison, WI, United States

**Keywords:** alemtuzumab, relapsing–remitting multiple sclerosis, prospective study, neurofilament light, glial fibrillary acidic protein

## Abstract

**Background:**

Alemtuzumab (ALZ) is a pulsed immune reconstitution therapy for multiple sclerosis (MS).

**Objective:**

To assess basic characteristics, therapeutic effects, and prognostic biomarkers on clinical and imaging parameters of disease activity for relapsing–remitting MS (RRMS) patients selected for ALZ, in a real-world long-term setting.

**Methods:**

Fifty-one RRMS patients [female = 31; mean age 36 (standard deviation 7.1) years; median expanded disability status scale (EDSS) 2 (interquartile range (IQR) 1.5)] initiating ALZ treatment, were consecutively included. Patients were assessed at baseline and thereafter annually for 5 years with clinical measures, symbol digit modality test (SDMT), and magnetic resonance imaging (MRI). Concentrations of glial fibrillary acidic protein (GFAP), reflecting astrogliosis, and neurofilament light (NfL), reflecting axonal damage, were measured in cerebrospinal fluid (CSF) and serum samples collected at baseline and after 2 years in CSF, and annually in serum. Control subjects were symptomatic controls (SCs, *n* = 27), who were examined at baseline and after 5 years without evidence of neurological disease.

**Results:**

While the mean annualized relapse rate was significantly reduced from baseline at each year of follow-up, disability was essentially maintained at a median EDSS of 1.5 and IQR between 1.13 and 2.25. New MRI activity was recorded in 26 patients (53%) over 5 years. The proportion of patients who achieved no evidence of disease activity (NEDA-3), 6-months confirmed disability worsening (CDW), and 6-months confirmed disability improvement (CDI) at 5 years were 33, 31, and 31%, respectively. The SDMT score was reduced for patients (*p* < 0.001), but unchanged for SCs. ALZ treatment did not change GFAP levels, whereas there was a significant decrease for RRMS patients in median CSF and serum NfL levels at follow-up [CSF month 24: 456 pg./mL (IQR 285.4) (*p* = 0.05); serum month 24: 6.7 pg/mL (IQR 4.7) (*p* < 0.01); serum month 60: 7.2 pg/mL (IQR 4.7) (*p* < 0.01)], compared to baseline [CSF: 1014 pg/mL (IQR 2832.5); serum 8.6 pg/mL (IQR 17.4)].

**Conclusion:**

In this real-world mono-center population, we observed a progression-free survival of 69%, cumulative NEDA-3 of 33%, and reduced NfL levels, over a five-year follow-up. This confirms ALZ as an effective pulsed immune reconstitution therapy that significantly reduces neuro axonal loss, and therefore has the potential to reduce long-term neurological disability. ALZ did not appear to affect astrogliosis.

## Introduction

Of the chronic inflammatory autoimmune diseases that affect the central nervous system (CNS), Multiple sclerosis (MS) is the commonest. Disease-modifying treatment (DMT) improves the course of the disease and prognosis (1). Two main treatment strategies are the maintenance-escalation approach and the pulsed immune reconstitution approach. The former means continuous medication which is escalated upon breakthrough disease activity, and the latter involves few treatment courses with long-term effects on disease activity (2).

The humanized monoclonal antibody alemtuzumab (ALZ) is one of the pulsed immune reconstitution therapies available today. ALZ mediates its effect by binding to CD52 (3) on the surfaces of T and B lymphocytes that harbor in the circulation. In less than a week, this leads to an extensive reduction of autoreactive T and B lymphocytes, thereafter an establishment of a new immune cell population will take place, which is less prone to cause an immunological attack to the CNS (4–7). The efficacy of ALZ on clinical and imaging parameters has been shown in one phase II (CAMMS223) (8), and two phase III (CARE-MS I & II) (9, 10) clinical trials, as well as in observational studies, together with patients with highly active disease (11–15).

ALZ has been accepted as treatment for relapsing–remitting MS (RRMS) in the European Union since 2013, and in the USA since 2014 (16). Due to pharmacovigilance data, ALZ underwent a review according to Article 20 of Regulation (EC) No 726/2004 in 2019, the results of which led to a label change (17). According to the latest recommendations from the European Medicines Agency, highly active RRMS patients with breakthrough disease activity in spite of ongoing DMT, or naïve patients with severe RRMS, can be treated with ALZ (18). ALZ is contraindicated in patients with specific heart, circulation, or hematological diseases, and in patients with other autoimmune diseases than MS.

Even if the efficacy of ALZ in the reduction of disease activity in RRMS has been widely demonstrated in three pre-marketing clinical trials (8–10), and two extension studies (12, 13), real-world data about the effectiveness of ALZ is limited (11), and no post-marketing randomized controlled trials have been conducted (19). Until now, results from to us known real-world studies (20–24) are in accordance with the discoveries of the clinical trials and their extensions. However, in these real-world studies, the mean follow-up period has been limited to 16–36 months, and in some of these studies common MS-related outcomes are missing. In this five-year prospective mono-center study, we therefore further extended the efficacy and safety data of ALZ, included all commonly used MS-related outcomes, and added data of glial fibrillary acidic protein (GFAP), reflecting astrogliosis, and neurofilament light (NfL), reflecting axonal damage, in cerebrospinal fluid (CSF) and serum.

## Materials and methods

### Patients and controls

RRMS patients, 18 years of age or more, fulfilling the 2017 revised McDonald diagnostic criteria of MS (25), were consecutively included in the study when they commenced ALZ treatment, at the MS Center, Sahlgrenska University Hospital, Gothenburg, between July 2014 and May 2016. Eligible patients for ALZ treatment may have been previously untreated or treated with one or several DMTs. They should not have ongoing acute or chronic infection, severe cardiovascular disease, stroke, coagulation disorders or treatments that affected coagulation, and were not allowed to have other autoimmune diseases than MS. Age and sex matched symptomatic controls (SCs) were recruited from persons with symptoms suspected for MS, referred to the MS Center, Sahlgrenska University Hospital, Gothenburg, between April 2014 and June 2016, and with negative diagnostic work-up (26). SCs were re-examined after five years. SCs were defined as having no history or signs of neurological disease, and no abnormal findings on imaging [magnetic resonance imaging (MRI)] or in the CSF analysis.

### Treatment

Patients received 60 mg intravenous (iv) ALZ on five consecutive days, and after 12 months, a repeated dose of 36 mg iv over three consecutive days. New courses of 36 mg iv ALZ were administered at signs of new disease activity, i.e., relapse, and/or detection of new, enlarging or contrast-enhancing MRI lesions. Premedication with 1000 mg iv methylprednisolone, oral antihistamine, paracetamol, and omeprazole was given the first three days of ALZ infusions. In addition, patients received prophylactic anti-herpesvirus treatment with acyclovir 200 mg twice daily for one month.

### Study design

**Figure 1:**
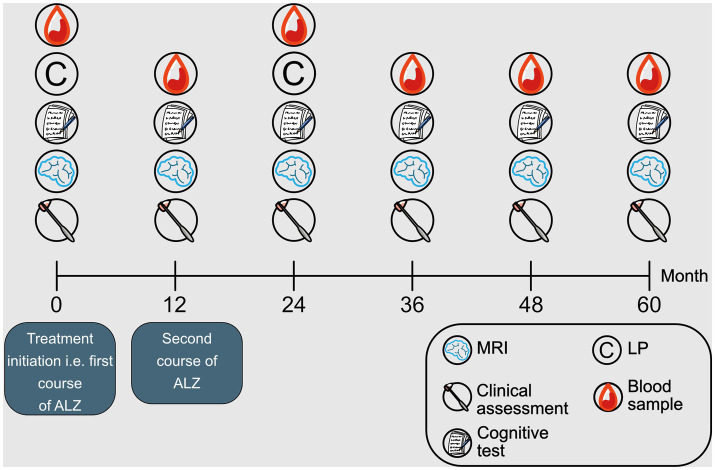
Study design. Schematic illustration of the study design. Month 0 (baseline). ALZ, alemtuzumab; MRI, magnetic resonance imaging; LP, lumbar puncture.

This was a prospective mono-center observational study with a follow-up of five years. Patients were assessed at month 0 (baseline), 12, 24, 36, 48 and 60 (Figure 1). Baseline was the date of initiating ALZ treatment. Patient baseline and follow-up outcomes were assessed by clinical evaluation of relapses, neurological examination with assessment of disability, cognitive test, MRI, CSF and serum concentrations of GFAP, and NfL. We also recorded the number of ALZ courses received per patient, durability of ALZ treatment, the proportion of patients who switched to another DMT, and adverse events.

### Clinical outcome measures

If new neurological symptoms appeared, or if old symptoms returned, for a period of 24 h or more, and no infection or raised core body temperature were present, this was categorized as a clinical relapse (27). Clinical activity was measured with the annualized relapse rate (ARR). Disability was assessed with the expanded disability status scale (EDSS) (28). Confirmed disability worsening (CDW) was achieved if an increase in the EDSS score occurred, relative to baseline, and was confirmed over 6 months. The threshold levels of change in EDSS to achieve CDW were as follows: an increase to 1.5 from a baseline EDSS of 0, an increase of 1 point from a baseline of 1–5.0, and an increase of 0.5 from a baseline EDSS score ≥ 5.5. Confirmed disability improvement (CDI) was achieved if a decrease in EDSS score occurred, relative to baseline, and was sustained for 6 months or more. To achieve CDI there had to be a decrease in EDSS score of ≥1 point if baseline EDSS score was 2.0–5.5, or a decrease of ≥0.5 points if baseline score was ≥6.0. No evidence of disease activity-3 (NEDA-3), was defined as absence of relapses, disability worsening, and new and/or enlarged T2 lesions, or gadolinium-enhancing lesions on MRI (29), while patients with either a relapse, MRI activity, or exacerbation of neurological disability, were considered as having evidence of disease activity-3 (EDA-3). Progression independent of relapses (PIRA) was defined as a 6-month CDW event with no previous or subsequent relapse during the five-year (60-month) follow-up period, whereas relapse associated worsening was defined as a 6-month CDW event with a previous relapse during follow-up.

### Cognitive test

Cognition was assessed with symbol digit modality test (SDMT) (30). To minimize learning bias a new version of SDMT was used at each time. We defined a clinically significant change in SDMT scores as an increase or decrease of eight or more raw score points from baseline, given that no reverse change in SDMT scores to less than in total eight points, compared with baseline, were observed during the following year (31). With a threshold of eight for SDMT, the following classification for RRMS patients were used: improvers (positive changes equal to or above the threshold level), maintainers (changes not reaching the threshold level) or decliners (negative changes equal to or above the threshold level).

### Safety

The common terminology criteria for adverse events (CTCAE), Version 5.0 (32), were used to grade adverse events. For early adverse events (<100 days after ALZ initiation), adverse events of grade 3 or higher were recorded. For late adverse events (>100 days after ALZ initiation) grade 2 or higher were recorded for autoimmune or infectious conditions, and grade 3 or higher for other adverse events.

### MRI

Brain MRI according to Swedish guidelines in MS (33) was performed, with a 3.0 Tesla MRI scanner. We obtained T1- and T2-weighted sequences, fluid-attenuated inversion recovery sequences, and gadolinium-enhanced T1 sequences. In the major part of cases contrast-enhancement on MRI is visible during a period of 6 weeks, mean 3.07 weeks (34). To interrelate MRI measures, and CSF and serum biomarker concentrations, we therefore included MRI performed 6 weeks prior to or after lumbar puncture and blood sampling. We defined new disease activity on MRI as occurrence of a new contrast-enhancing lesion or new or enlarged T2 lesions, compared to preceding MRI.

### Biomarker analysis

Samples of CSF were obtained at month 0 and 24, and serum samples from peripheral blood were obtained at month 0, 12, 24, 36, 48, and 60. The consensus protocol of the BioMS-EU network for CSF biomarker research in MS was followed (35). In short CSF samples were collected and transferred to polypropylene tubes for centrifugation at 20°C, 2200 *g* for 10 min. Samples were then distributed into 1 mL aliquots in polypropylene vials, and permanently stored at −80°C pending analyses. Blood samples were collected, processed onsite to isolate serum, aliquoted, and frozen at −80°C. All biomarker analyses were performed by board-certified laboratory technicians in the Clinical Neurochemistry Laboratory at the Sahlgrenska University Hospital. To account for potential variation between plates, baseline and follow-up samples were analyzed side-by-side on the same assay plate. All analyses were performed at room temperature.

The analyses of CSF and serum biomarkers (NfL, and GFAP) were performed using the Simoa^®^ NEUROLOGY 2-PLEX B Kit, Product number: 103520, from Quanterix (Billerica, MA, USA). Briefly, the samples, quality control (QC) samples, with one QC high (within the higher measurement interval) and one QC low (within the lower measurement interval), and a set of pre-mixed calibrator samples (Product number: 103520, Quanterix, Billerica, MA, USA) were removed from storage and allowed to thaw at room temperature. The resorufin-D-galactopyranoside reagent was shaken for 30 min at 800 rpm and 30°C. The samples, QCs, and calibrators were vortexed for 30 s at 2000 rpm. The samples and QCs were thereafter centrifuged for 10 min at 4000 *g*. CSF samples were diluted 40x (10x pre-dilution +4x on-board), and serum samples 4x (on-board), both with sample dilution reagent. The samples, QCs, and calibrators were added to a Simoa plate, the calibrators right after a throughout mixing. The assay reagents and a Simoa plate with samples, QCs, and calibrators were loaded in a HD-1 Analyzer (Quanterix, Lexington, MA, USA), the beads after a throughout mixing for 30 s, and the measurements initiated. Intra- and inter assay coefficients of variation were below 10% for both QC high and QC low, for both GFAP and NfL. The functional Lower Limit of Quantification (fLLoQ) for serum GFAP and NfL was 29.4 pg/mL, respectively, 1.41 pg/mL. The fLLoQ for CSF was 10x the fLLoQ for serum. The CSF and serum samples of GFAP and NfL below the fLLoQ level were designated the value of fLLoQ.

### Statistical analysis

Descriptive statistical analyses were used to analyze data. Continuous variables are expressed as the median and interquartile range (IQR), mean and standard deviation (SD), or mean and 95% confidence interval (CI). Categorical variables are expressed as the frequency and percentage. As the data were not normally distributed, non-parametric tests were used. Wilcoxon matched-pair singed-rank test was used to evaluate changes in continuous variables within groups, between two measured times. Mann–Whitney *U* test was used to compare continuous variables between groups. Fischer’s exact test was used to compare categorical variables between groups. Cumulative probabilities of NEDA-3, CDW, and CDI were estimated with Kaplan–Meier curves. To assess the impact of baseline variables (age, gender, diagnosis duration, EDSS, and CSF NfL) on the outcomes of fulfilling EDA-3, CDW, and CDI at five-year follow-up, Kaplan–Meier survival analyses with corresponding log-rank tests were performed. For these analyses we used categorical nominal variables of age, diagnosis duration, EDSS, and CSF NfL. The cut-off for age (≤30 years) was based on previous reports regarding a significant change in the relationship between disability and onset age after about 30 years (36, 37). Diagnosis duration was divided into three groups: ≤3 years, >3 to <10 years, and > 10 years, and EDSS into two groups: <3, and ≥ 3. The cut-off (>2136 pg/mL) for CSF NfL was based on calculation of the third of patients with the highest CSF NfL. For statistical analyses SPSS version 28.00 (IBM, NY, US) and GraphPad Prism 10.0.2 (GraphPad Inc., California, USA) were used. All tests were two-sided, with a significance threshold of *p* ≤ 0.05.

## Results

### Baseline characteristics of patients and controls

We included 51 RRMS patients, 31 females, mean age 36 years (SD 7.1) in the study: 6 (12%) were treated with first-line DMTs, 38 (74%) had second-line DMTs, and 7 (14%) were treatment naïve. The most common reasons for initiating ALZ treatment were breakthrough disease (45%), increased John Cunningham (JC) virus antibody index in natalizumab treated patients (35%), and highly active disease from disease onset (14%). Baseline demographics and clinical characteristics of RRMS patients, and SCs are presented in Table 1. All patients were followed for five years, except two patients that were lost to follow-up, one due to change of residence, and one due to breast cancer.

**Table 1:** Baseline characteristics of RRMS patients, and SCs.

Recorded baseline features	Population		
	RRMS	SCs	*p*-value
	(*n* = 51)	(*n* = 27)	
Age years, mean (SD)	36 (7.1)	35.3 (10)	0.80
Gender, female, n (%)	31 (61%)	21 (78%)	0.20
Diagnosis duration, years, mean (SD)	5.7 (5.2)		
EDSS median (IQR)	2 (1.5)		
ARR the year before ALZ, mean (SD)	0.49 (0.68)		
Treatment prior to baseline			
First-line (IFNB, GLA, DMF, TFL), n (%)	6 (12%)		
Second-line (NZB, FGL, RITX), n (%)	38 (74%)		
Naive, n (%)	7 (14%)		
Reasons to switch to ALZ			
Breakthrough disease activity despite DMT, n (%)	23 (45%)		
Adverse event, n (%)	3 (6%)		
Positive JC virus test during NZB treatment, n (%)	18 (35%)		
Highly active disease from disease onset, n (%)	7 (14%)		
MRI at baseline			
T2 lesions 1–9, n (%)	4 (8)		
T2 lesions 10–20, n (%)	12 (23)		
T2 lesions >20, n (%)	35 (69)		
Number of patients with contrast-enhancing lesions, n (%)	15 (29)		
Number of patients who received each course of ALZ			
First course, n (%)	51 (100%)		
Second course, n (%)	50 (98%)		
Third course, n (%)	17 (33%)		
Fourth course, n (%)	2 (4%)		

RRMS, relapsing–remitting multiple sclerosis; SCs, symptomatic controls; SD, standard deviation; EDSS, expanded disability status scale; ARR, annualized relapse rate; ALZ, alemtuzumab; IFNB, interferon beta; GLA, glatiramer acetate; DMF, dimethyl fumarate; TFL, teriflunomide; NZB, natalizumab; FGL, fingolimod; RITX, rituximab; DMT, disease-modifying treatment; JC virus, John Cunningham virus; MRI, magnetic resonance imaging; IQR, interquartile range.

Baseline disease activity of treatment naïve patients differed from those with previous DMT by statistically significant higher ARR [treatment naïve: mean ARR 0.86 (SD 0.38); previous DMT: mean ARR 0.42 (SD 0.70), *p* = 0.045], and higher proportion with contrast-enhancing lesions on MRI [treatment naïve: 100% (7/7); previous DMT: 18% (8/44), *p* < 0.001].

### The effect of alemtuzumab on relapses and MRI activity

**Figure 2:**
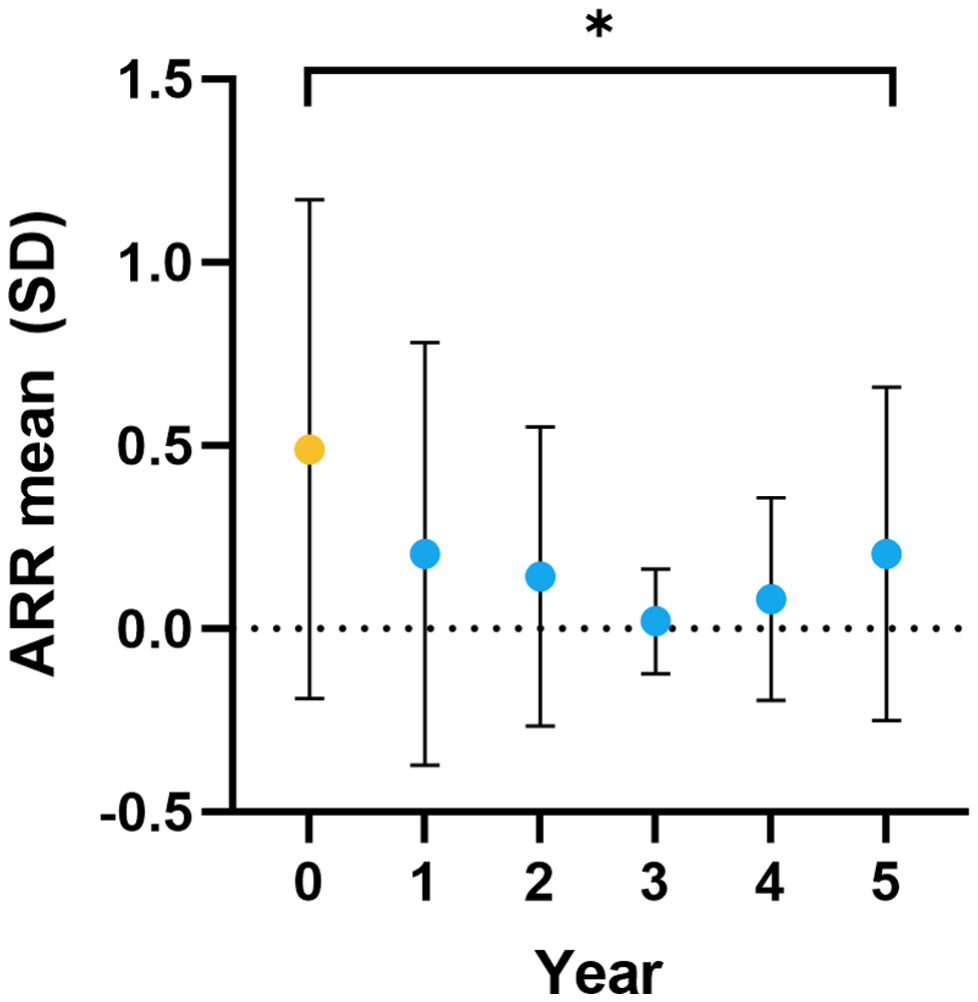
ARR over five years. ARR at baseline (yellow dot), i.e., the year before ALZ initiation (year 0), and at year 1–5 (blue dots) of follow-up. The dots represent mean values, and error bars represent the SD. ARR, annualized relapse rate; ALZ, alemtuzumab; SD, standard deviation. *p ≤ 0.05.

**Figure 3:**
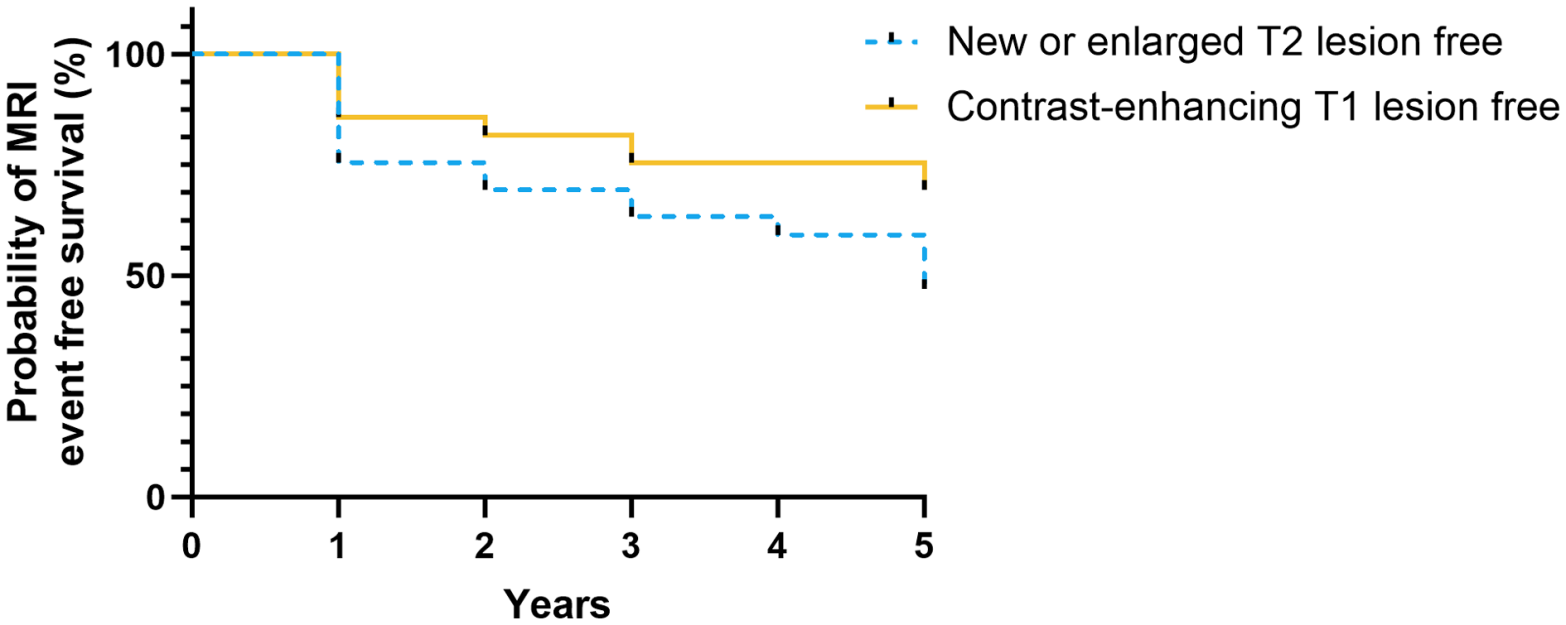
MRI outcomes. Kaplan–Meier plot shows probability of MRI event free survival. Yellow solid line represents the probability of contrast-enhancing T1 lesion free MRI, and blue dashed line represents the probability of new or enlarged T2 lesion free MRI, during follow-up. MRI, magnetic resonance imaging.

After commencing ALZ treatment, mean ARR was significantly reduced from baseline [0.49 (SD 0.68)] at each year of follow-up, with a mean ARR of 0.20 (SD 0.46), *p* < 0.05, at the five-year follow-up (Figure 2). At year five, 29 (60%) patients were still relapse free. New MRI activity (new or enlarged T2 lesions, and/ or contrast-enhancing T1 lesions) was recorded in 26 patients (53%) over five years. The number of patients with new or enlarged T2 lesions and contrast-enhancing T1 lesions during the study period was 26 (53%) and 15 (30%), respectively (Figure 3).

Breakthrough disease activity was recorded in 67% (33/49) patients, 52% (17/33) of those had a third course of ALZ, 6% (2/33) had a fourth course of ALZ, and 40% (13/33) switched from ALZ to another DMT (rituximab *n* = 8, autologous hematopoietic stem cell transplantation *n* = 3, natalizumab *n* = 2). At the five-year follow-up 73% (36/49) of patients were still treated on ALZ and 33% (16/49) were free from disease activity.

### The effect of alemtuzumab on disability

At baseline the median EDSS was 2.0 (IQR 1.5), and median EDSS was essentially unchanged during follow-up with a median EDSS of 1.5 and an IQR between 1.13 and 2.25. At year five 20% (10/49) patients had improved (≥1.0-point decrease), 53% (26/49) stable (≤0.5-point change), and 27% worsened (≥1.0-point increase) EDSS, compared to baseline. The Kaplan–Meier estimates of NEDA-3, 6-months CDW, and 6-months CDI at five years were 33, 31, and 31%, respectively. PIRA, and relapse associated worsening were observed in 6 (12%), respectively 9 (18%) patients over five years.

### The effect of alemtuzumab on cognition

**Figure 4:**
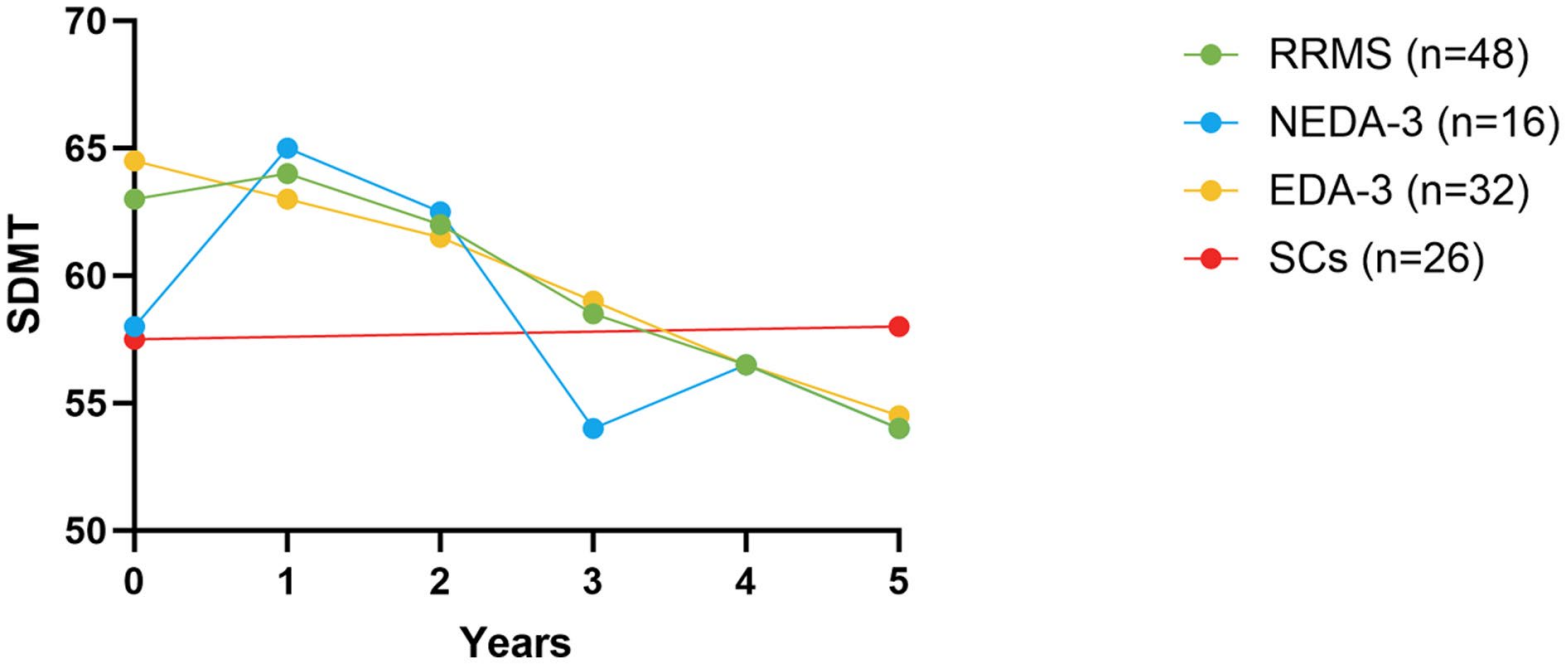
SDMT scores over five years. SDMT scores at baseline (i.e., year 0) and at year 1–5 of follow-up for RRMS patients (green dots), as well as for the NEDA-3 (blue dots) and EDA-3 (yellow dots) subgroups. For SCs (red dots) SDMT scores at baseline and year five are shown. The dots represent mean values. SDMT, symbol digit modality test; RRMS, relapsing–remitting multiple sclerosis; SCs, symptomatic controls; NEDA-3, no evidence of disease activity-3; EDA-3, evidence of disease activity.

At baseline, SDMT showed no statistically significant differences in cognition between RRMS patients and SCs (*p* = 0.051), or between the NEDA-3 and the EDA-3 subgroups (*p* = 0.52). However, SDMT scores were statistically significantly worse over five years for patients (*p* < 0.001), regardless whether they achieved NEDA-3 (*p* < 0.05), or had EDA-3 (*p* < 0.001), while the median SDMT score for SCs was essentially unchanged (Figure 4). Accordingly, at year five of follow-up, the median SDMT score was higher in SCs [58 (IQR 11.5), *n* = 26, *p* = 0.03] compared to patients [54 (IQR 12.75), *n* = 48]. Thus, disease activity or increasing disability during the study period seemed not to influence cognition since no significant differences were found between SDMT scores of the NEDA-3 and the EDA-3 subgroups at follow-up. However, if we applied the established clinical cut-off score for SDMT change (≥8 points) (31), 63% (20/32) of EDA-3 patients reduced their SDMT score, whereas only 31% (5/16) of NEDA-3 patients had worsened in SDMT (*p* = 0.048). Using this cut-off limit for clinically significant SDMT change 6% (*n* = 3) of patients were categorized as improvers, 42% (*n* = 20) as maintainers, and 52% (*n* = 25) as decliners.

### The effect of alemtuzumab on NfL and GFAP levels

A high proportion of patients and SCs refrained from lumbar puncture. At baseline, we had access to CSF and serum samples from 37 and 41 patients, and 25 and 27 SCs, respectively. In one patient, only CSF GFAP, and not CSF NfL, was analyzed.

**Figure 5:**
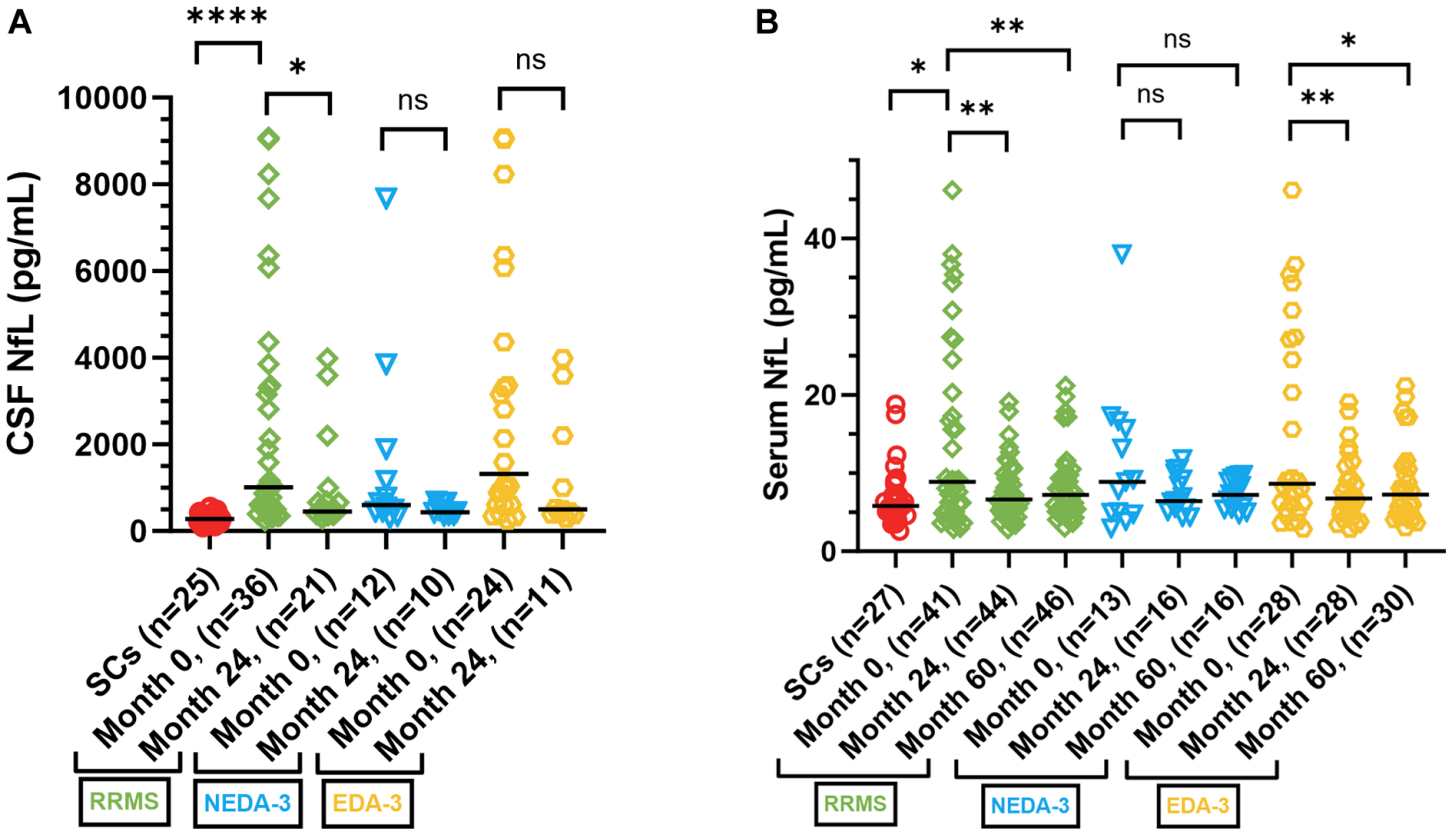
CSF and serum NfL levels in RRMS patients and SCs. **(A)** CSF NfL levels in SCs (red dots), RRMS patients (green squares), as well as in NEDA-3 (blue triangles) and EDA-3 (yellow hexagons) subgroups, at baseline (month 0), and at re-sampling at 24 months after treatment initiation. The scatter plot represent individual values, with a line at median. **(B)** Serum NfL levels in SCs (red dots), RRMS patients (green squares), as well as in NEDA-3 (blue triangles) and EDA-3 subgroups (yellow hexagons), at baseline (month 0), and at re-sampling at 24, and 60 months after treatment initiation. The scatter plot represent individual values, with a line at median. CSF, cerebrospinal fluid; RRMS, relapsing–remitting multiple sclerosis; SCs, symptomatic controls; GFAP, glial fibrillary acidic protein; NfL, neurofilament light; NEDA-3, no evidence of disease activity-3; EDA-3, evidence of disease activity; ns, not statistically significant. *p ≤ 0.05, **p ≤ 0.01, ****p ≤ 0.0001.

At baseline, patients had significantly higher median NfL levels in CSF [1014 pg/mL (IQR 2832.5), *p* < 0.0001] and serum [8.9 pg/mL (IQR 17.4), *p* = 0.03], compared to SCs [CSF: 281 pg/mL (IQR 156); serum: 5.8 pg/mL (IQR 3.8)] (Figures 5A,B). There was a significant decrease in median CSF (*p* = 0.05) and serum (*p* = 0.006) NfL levels of patients at two-year (24-month) follow-up, compared to baseline, and this reduction in serum NfL was maintained throughout the study (Figures 5A,B). In the subgroup analysis (Figures 5A,B), a significant decrease during follow-up was only found in serum NfL in the EDA-3 cohort. There was no significant difference in serum NfL levels between the NEDA-3 and EDA-3 subgroups.

**Figure 6:**
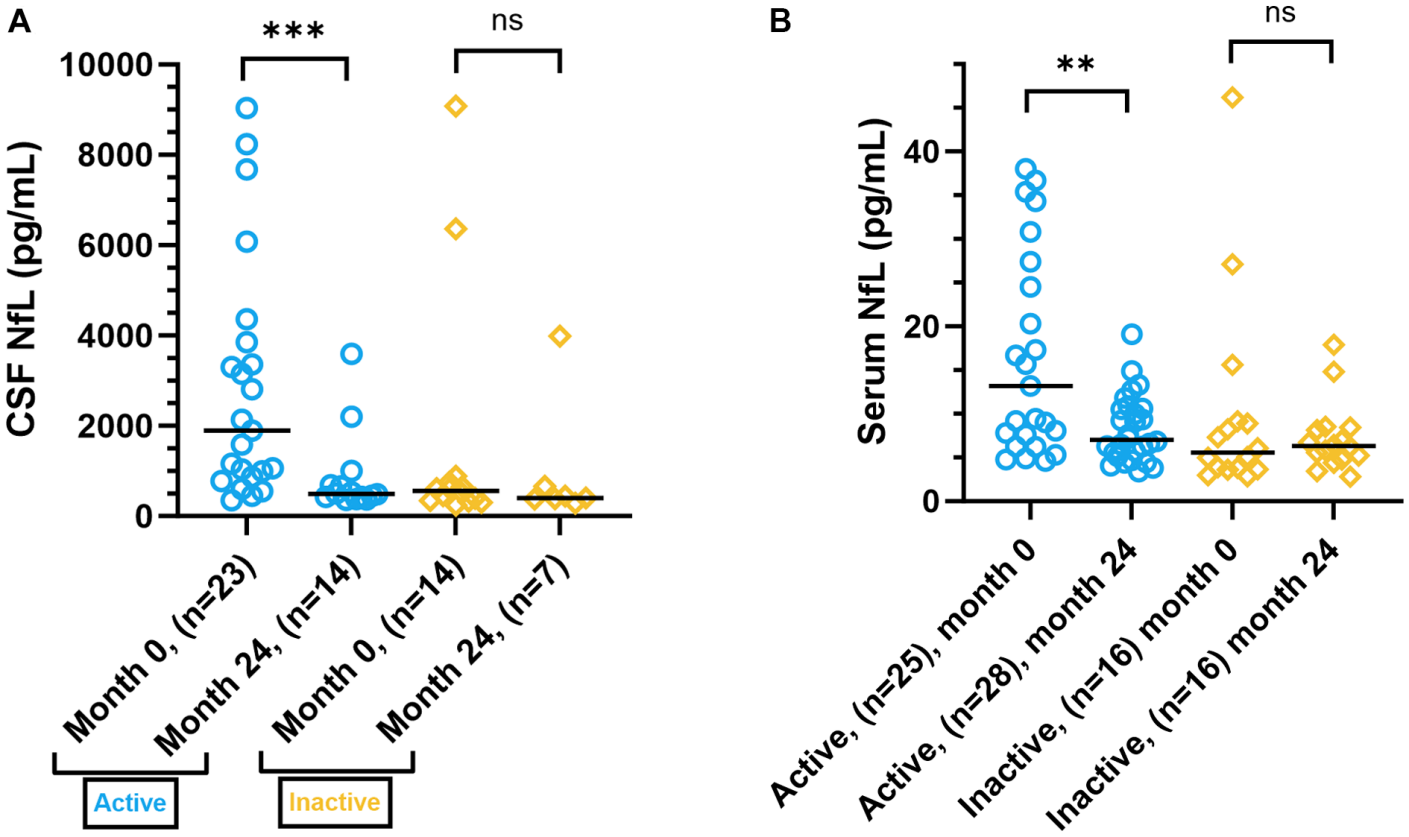
CSF and serum NfL levels in patients, grouped by reason for initiating ALZ. **(A)** CSF NfL levels in active (blue dots) patients (treatment naïve and breakthrough disease activity despite DMT), and inactive (yellow squares) patients (natalizumab treated patients with increased JC virus antibody index or patients with an adverse event on other DMT), at baseline (month 0), and at re-sampling at 24 months after treatment initiation. The scatter plot represent individual values, with a line at median. **(B)** Serum NfL levels in active (blue dots) and inactive (yellow squares) patients, at baseline (month 0), and at re-sampling at 24 months after treatment initiation. The scatter plot represent individual values, with a line at median. CSF, cerebrospinal fluid; NfL, neurofilament light; ALZ, alemtuzumab; DMT, disease-modifying treatment; ns, not statistically significant. **p ≤ 0.01, ***p ≤ 0.001.

We grouped patients according to their reason for initiating ALZ into active RRMS (treatment naïve and breakthrough disease activity despite DMT), and inactive patients (natalizumab treated patients with increased JC virus antibody index or patients with an adverse event on other DMT). At baseline, active patients had significantly higher median NfL levels in CSF [1892 pg/mL (IQR 2980), *n* = 23, *p* = 0.003] and serum [13.2 pg/mL (IQR 22.2), *n* = 25, *p* = 0.006], compared to inactive patients [CSF: 560 pg/mL (IQR 450.5), *n* = 14; serum: 5.6 pg/mL (IQR 5.4), *n* = 16]. At two-year follow-up, CSF (*p* = 0.0002) and serum (*p* = 0.002) NfL levels were significantly reduced in active patients, while NfL levels remained low in inactive patients (Figures 6A,B).

At baseline, patients had significantly higher median CSF GFAP levels [14720 pg/mL (IQR 9240), *n* = 37, *p* < 0.0001] than age-matched SCs [5189 pg/mL (IQR 2954), *n* = 25]. Similar differences were not found for serum GFAP. Disease activity and ALZ treatment did not appear to affect CSF nor serum GFAP concentrations. Repeated determinations of GFAP in CSF and serum showed similar levels in NEDA-3 and EDA-3 subgroups and ALZ treatment had no effect on GFAP concentrations in CSF or in serum. Furthermore, CSF and serum GFAP levels were similar in patients who achieved CDW or PIRA compared with those who did not.

Individual serum NfL and GFAP concentrations were determined at baseline, and during follow-up, for each patient who had a baseline value (Supplementary Figures 1A,B). Serum NfL levels remained low after ALZ treatment while GFAP levels appeared unaffected and had a greater intra- and inter-individual variability.

### The prognostic value of demographic, baseline clinical characteristics and biomarkers on clinical outcomes

To assess the prognostic value of baseline variables (age, gender, diagnosis duration, EDSS, and CSF NfL) on the outcomes of fulfilling EDA-3, CDW, and CDI at five-year follow-up, Kaplan–Meier survival analyses with corresponding log-rank tests were performed. The results from these analyses are presented in Table 2.

**Table 2:** The prognostic value of demographic, baseline clinical characteristics and biomarkers, on clinical outcomes at five-year follow-up.

Variable	Category	All patients			Active patients		Inactive patients			
		No. of patients Events;Total	Mean survival time y (95% CI)	*p*-value	No. of patients Events;Total	Mean survival time y (95% CI)	*p*-value	No. of patients Events;Total	Mean survival time y (95% CI)	*p*-value
Outcome EDA-3										
Gender	Female	21;30	3.0 (2.4, 3.7)	0.55	9;14	2.8 (1.8, 3.7)	0.80	12;16	3.3 (2.4, 4.1)	0.54
	Male	12;19	3.3 (2.5, 4.2)		10;15	3.3 (2.4, 4.3)		2;4	3.3 (1.5, 5.0)	
Age	≤30 y	11;11	1.9 (0.9, 2.9)	<0.001	5;5	1.0 (1.0, 1.0)	<0.001	6;6	2.7 (1.1, 4.2)	0.09
	>30 y	22;38	3.5 (2.9, 4.1)		14;24	3.5 (2.8, 4.2)		8;14	3.5 (2.6, 4.4)	
Diagnosis duration	≤3 y	17;25	3.1 (2.4, 3.9)	0.66	11;17	3.1 (2.2, 4.0)	0.84	6;8	3.1 (1.7, 4.6)	0.75
	>3- < 10 y	8;11	2.6 (1.5, 3.8)		4;6	2.3 (0.8, 3.8)		4;5	3.0 (1.2, 4.8)	
	≥10 y	8;13	3.6 (2.7, 4.5)		4;6	3.7 (2.2, 5.1)		4;7	3.6 (2.5, 4.7)	
EDSS	<3	26;38	3.0 (2.4, 3.6)	0.56	14;22	3.0 (2.2, 3.8)	0.86	12;16	3.0 (2.1, 3.9)	0.28
	≥3	7;11	3.6 (2.6, 4.7)		5;7	3.3 (1.8, 4.7)		2;4	4.3 (2.5, 6.1)	
CSF NfL	≤2136 pg/mL	12;22	3.6 (2.8, 4.3)	0.05	6;12	3.8 (2.8, 4.9)	0.07	6;10	3.2 (1.9, 4.5)	0.44
	>2136 pg/mL	10;12	2.3 (1.3, 3.2)		8;10	2.1 (1.2, 3.0)		2;2	3.0 (0.0, 6.9)	
Outcome CDW										
Gender	Female	11;30	4.0 (3.5, 4.5)	0.19	6;14	3.6 (2.7, 4.5)	0.11	5;16	4.4 (3.8, 4.9)	0.87
	Male	4;19	4.7 (4.5, 5.0)		3;15	4.8 (4.6, 5.0)		1;4	4.5 (3.7, 5.4)	
Age	≤30 y	5;11	3.5 (2.5, 4.6)	0.13	4;5	2.6 (3.1, 5.5)	0.002	1;6	4.3 (3.1, 5.5)	0.50
	>30 y	10;38	4.5 (4.2, 4.8)		5;24	4.5 (4.1, 5.0)		5;14	4.4 (4.0, 4.9)	
Diagnosis duration	≤3 y	8;25	4.0 (3.4, 4.6)	0.74	6;17	3.9 (3.2, 4.7)	0.65	2;8	4.3 (3.3, 5.2)	0.95
	>3- < 10 y	4;11	4.5 (3.9, 5.0)		2;6	4.3 (3.5, 5.2)		2;5	4.6 (4.2, 5.0)	
	≥10 y	3;13	4.6 (4.2, 5.0)		1;6	4.8 (4.5, 5.1)		2;7	4.4 (3.9, 4.9)	
EDSS	<3	10;38	4.4 (4.1, 4.8)	0.16	7;22	4.2 (3.6, 4.8)	0.86	3;16	4.8 (4.6, 5.0)	0.001
	≥3	5;11	3.7 (2.8, 4.7)		2;7	4.3 (3.3, 5.3)		3;4	2.8 (1.3, 4.2)	
CSF NfL	≤2136 pg/mL	7;22	4.5 (4.1, 4.8)	0.76	4;12	4.4 (3.9, 5.0)	0.96	3;10	4.5 (3.9, 5.1)	0.38
	>2136 pg/mL	4;12	3.9 (3.0, 4.9)		3;10	4.1 (3.1, 5.1)		1;2	3.0 (0.3, 5.8)	
Outcome CDI										
Gender	Female	10;30	3.8 (3.2, 4.4)	0.61	4;14	*	0.83	6;16	*	0.17
	Male	5;19	4.1 (3.3, 4.8)		5;15	*		0;4	*	
Age	≤30 y	4;11	3.5 (2.4, 4.7)	0.60	1;5	4.2 (2.8, 5.6)	0.58	3;6	3.0 (1.4, 4.6)	0.15
	>30 y	11;38	4.0 (3.5, 4.5)		8;24	3.8 (3.0, 4.5)		3;14	4.4 (3.8, 5.0)	
Diagnosis duration	≤3 y	11;25	3.5 (2.8, 4.2)	0.12	6;17	*	0.72	5;8	*	0.04
	>3- < 10 y	1;11	4.6 (4.0, 5.3)		1;6	*		0;5	*	
	≥10 y	3;13	4.1 (3.2, 5.0)		2;6	*		1;7	*	
EDSS	<3	9;38	4.2 (3.7, 4.7)	0.04	4;22	4.3 (3.7, 4.9)	0.006	5;16	4.0 (3.2, 4.8)	0.85
	≥3	6;11	2.9 (1.8, 4.0)		5;7	2.3 (1.0, 3.6)		1;4	4.0 (2.3, 5.7)	
CSF NfL	≤2136 pg/mL	4;22	4.4 (3.8, 4.9)	0.05	1;12	4.7 (4.0, 5.3)	0.03	3;10	4.0 (3.0, 5.0)	0.56
	>2136 pg/mL	6;12	3.1 (2.0, 4.2)		5;10	3.1 (1.9, 4.3)		1;2	3.0 (0.3, 5.8)	

* No statistics computed because all cases censored. No., number; CI, confidence interval; EDA-3, evidence of disease activity; CSF, cerebrospinal fluid; NfL, neurofilament light; EDSS, expanded disability status scale; CDI, confirmed disability improvement; CDW, confirmed disability worsening; y, years.

**Figure 7:**
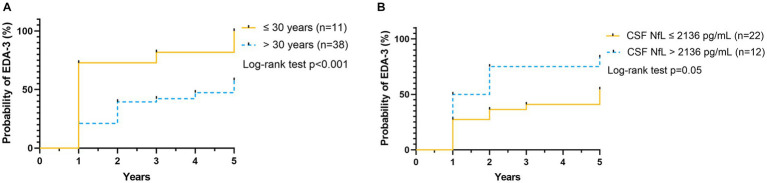
Probability of EDA-3, relative to age and CSF NFL. Kaplan–Meier plot shows the probability of RRMS patients to reach EDA-3 at the indicated time (years), according to baseline variables **(A)** age ≤ 30 years (*n* = 11) represented by yellow solid line, or age > 30 years (*n* = 38) represented by blue dashed line, and **(B)** CSF NfL ≤ 2,136 pg/mL (*n* = 22) represented by yellow solid line, or CSF NfL > 2136 pg/mL (*n* = 12) represented by blue dashed line. RRMS, relapsing–remitting multiple sclerosis; EDA-3, no evidence of disease activity-3; CSF, cerebrospinal fluid; NfL, neurofilament light.

Gender did not appear to affect the likelihood of meeting criteria for EDA-3, CDW, or CDI at follow-up (Table 2). In the entire patient population (*p* < 0.001) and in the subgroup of patients classified as active (*p* < 0.001) young age (≤30 years) appeared to indicate higher probability of showing signs of disease activity (EDA-3) at follow-up, compared to patients with an older age (>30 years) (Figure 7A and Table 2). For active patients, young age also appeared to indicate higher likelihood of exhibiting with CDW (*p* = 0.002) at follow-up, compared to older patients, this was not seen for the entire patient population or inactive patients (Table 2).

When looking at the entire patient population and the subgroup of patients classified as active, duration of diagnosis did not appear to affect the likelihood of encountering EDA-3, CDW or CDI at follow-up (Table 2). However, for inactive patients, diagnosis duration appeared to affect the probability of reaching CDI (*p* = 0.04), with a probability of 63%, 0%, respectively 14%, for patients with diagnosis duration ≤3 years, >3 to <10 years, respectively >10 years. In addition, patients with EDSS <3 were significantly less likely to reach CDI at follow-up (*p* = 0.04), compared to patients with EDSS ≥3, this was also true for the subgroup of active patients (*p* = 0.006), but not for the inactive subgroup (Table 2).

In terms of biomarkers, high CSF NfL (CSF NfL > 2136 pg/mL) indicated higher likelihood of meeting EDA-3 at follow-up [mean survival time 2.3 years (95% CI 1.3, 3.2), *p* = 0.05], compared to CSF NfL ≤ 2136 pg/mL [mean survival time 3.6 years (95% CI 2.8, 4.3)] (Figure 7B), this was true for the entire patient population, but not for the active and inactive subgroups (Table 2). On the other hand, for those patients who achieved CDI during follow-up, we could see that those with higher NfL achieved CDI earlier (*p* = 0.05), compared to those with lower NfL, this also applied to the subgroup of active patients (*p* = 0.03), but not to the inactive subgroup (Table 2).

### Safety

The included patients in this study have previously constituted a smaller part of a larger population where safety regarding ALZ treatment has been described (38).

None of the patients had an adverse event of CTCAE grade 3 or higher throughout the first 100 days after ALZ initiation. Twenty-nine patients (59%) developed autoimmune adverse events. There was no mortality in the patient group. See Table 3 for more detailed information on the safety outcomes.

**Table 3:** Safety.

Adverse event	RRMS patients (*n* = 49)
First 100 days after ALZ initiation	
CTCAE grade 3 or higher, n (%)	0 (0%)
Late adverse events (>100 days after ALZ initiation)	
CTCAE grade 3 or higher, n (%)	4 (8%)
ITP	3 (6%)
Skin cancer	1 (2%)
CTCAE grade 2, n (%)	
Autoimmune thyroid disease	27 (55%)
Neutropenia (autoimmune)	2 (4%)
Herpes zoster infection	3 (6%)
Upper respiratory infection	2 (4%)
Urinary tract infection	2 (4%)

RRMS, relapsing–remitting multiple sclerosis; ALZ, alemtuzumab; CTCAE, the common terminology criteria for adverse events; ITP, immune mediated thrombocytopenia.

## Discussion

In this prospective mono-center study, of real-world ALZ treated RRMS patients, we observed during five-years of follow-up, a reduction of ARR, stabilization of EDSS, a progression-free survival of 69%, freedom of MRI activity in 47, and 33% achieved NEDA-3. However, ALZ did not appear to affect further deterioration in cognition (SDMT). While NfL concentrations were reduced in CSF and serum, GFAP concentrations were essentially unchanged. Further, we observed that low age and high CSF NfL levels at baseline were negative prognostic markers for achieving NEDA-3 at the five-year follow-up, while EDSS <3 at baseline was a negative prognostic marker for achieving CDI.

We observed a significant reduction in ARR over each of year 1–5 of the follow-up, and a low MRI activity, which is consistent with what was reported from two phase III clinical trials (9, 10), their five-year extensions (12, 13), as well as real-world studies (20–24) of ALZ in RRMS. However, we could not confirm a clear tendency or a statistically significant reduction in T1 contrast-enhancing lesions during follow-up. This was probably an effect of including stable JC virus antibody positive natalizumab treated patients in our study cohort. They comprised more than one-third of patients, and only 29% of our patients had T1 contrast-enhancing lesions at baseline, compared with 46% (9), 42% (10) and 37% (23) in other studies.

We observed no change in median EDSS, during the five-year follow-up period. Although EDSS stabilized, only 20% had improved (EDSS ≥1.0 point decrease). Our result contrast with three similar real-world studies (20–22), showing significant reductions in EDSS. This discrepancy may be explained by a lower baseline median level of EDSS (2.0), than in these studies [4.0 (20), 3.0 (21), respectively 2.5 (22)], and a longer follow-up period (five years), than in these studies [36 months (20), 16 months (21), respectively 24 months (22)]. A more established measure of change in disability is 6-month CDW and CDI. ALZ was the first DMT to show CDI (8). Of our patients, 31% achieved CDI during follow-up compared with 33% (12) respectively 43% (13) of patients in the five-year extensions of the two phase III clinical trials of ALZ. Thus, despite having a large stable previously natalizumab treated patient group, we reached almost the same proportion of CDI as in the extension of CARE-MS I (12), which only included untreated RRMS patients.

At five-year follow-up, 33% of patients among our ALZ treated cohort achieved NEDA-3. This was slightly higher compared to the extension study of the CARE-MS II trial, where 27% of patients achieved NEDA-3 at year five (13), but slightly lower than in the CARE-MS I trial, in which 40% of patients achieved NEDA-3 at year five (12). These differences are probably due to differences in the selection of study populations with the highest proportion of treatment naïve patients in the CARE-MS I trial and the lowest in the CARE-MS II trial. Two real-world studies (20, 22) of ALZ, have reported even higher NEDA-3 achievements, with 45% (20), and 43.7% (22), respectively. However, their follow-up time, 36 months (20) and 24 months (22), respectively, was shorter than our (60 months).

At baseline, no statistically significant difference was observed between SDMT scores obtained from SCs and RRMS patients, but during follow-up a significant decrease developed for RRMS patients, which also included patients who achieved NEDA-3. However, if we applied the clinical cut-off limit of SDMT change of eight or more raw score points, a significant impairment of information processing speed was observed only in the EDA-3 and not in the NEDA-3 subgroup. We recorded a mean reduction in the SDMT score of 11.6 points after five-years of follow-up. This was in contrast with two previous real-world studies, which reported mean improvements in the SDMT score of 4.3 (39) and 5.2 (40) points. However, their results were obtained after only 15, respectively, 24 months of ALZ treatment, respectively. The cognitive decline in the current study occurred after five-years from commencing ALZ treatment, and was not observed in studies with shorter follow-up (39, 40). Thus, the rate of cognitive decline seemed slow and associated with disease activity (EDA-3).

We observed statistically significant higher levels of GFAP and NfL in patients at baseline, compared to SCs. Although NfL is considered a biomarker of axonal damage, it mostly reflects disease activity in MS (41). ALZ treatment reduced NfL levels of the entire patient population, to levels of SCs, and they remained at low levels during follow-up. In line with previous studies (42–45) the NfL reduction was limited to patients with disease activity at baseline. In contrast, treatment with ALZ did not influence GFAP levels. Similar unresponsiveness of GFAP to DMTs has been reported previously (42, 43). However, while serum NfL levels appeared stable during follow-up, the GFAP levels showed a larger intra- and inter-individual variation. This may imply that processes affecting astrocyte activation and astrogliosis are not influenced by ALZ treatment. We have previously shown that GFAP is associated with disability and is particularly increased during progressive MS (42, 43). However, we did not observe that GFAP levels increased in patients that achieved CDW or PIRA, but this may be due to the limited number of patients who showed disability progression at follow-up.

Among all demographic, clinical characteristics, MRI and fluid biomarkers at baseline only high CSF NfL levels and age could predict outcome in the current RRMS population. The prognostic value of NfL has been shown previously in several studies (46–48), but the negative predictive value on NEDA-3 from low age (≤30 years) at baseline was more unexpected. Although younger age at onset is associated with increased relapse rate and lower rate of disability progression (49), the impact from onset age on prognosis has been questioned (36, 37). In our study population, patients with younger age had, not unexpected, shorter disease duration than older patients. Despite shorter disease duration, they showed as high T2 lesion burden as older patients at baseline, which may indicate a higher disease activity and thus worse prognosis. However, the understanding of how age of onset affects the degree of accrued neurological disability in MS patients is not particularly understood. To advance the knowledge of the prognostic value of onset age, there are a need for further studies.

The main limitation of our study was the use of a real-world setting with a more heterogeneous population, probably less accurate clinical evaluations and missing examinations and tests at follow-up. In addition, the EDSS is widely known to have a high interrater variability (50), as does the interrater variability of MRI lesion assessment (51). However, because all patients were assessed at a single center and mostly by the same experienced neurologists and radiologists, this variation may have been limited. Another limitation was a small patient sample size (51 RRMS patients).

To our knowledge this was the first real-world prospective study on ALZ treatment in RRMS with a five-year follow-up. In contrast with most other DMTs, ALZ is a pulsed immune reconstitution therapy, administered iv with two or only few additional courses. We confirmed that ALZ is highly effective and has long-term effects on relapse rate and MRI outcomes and appeared to stabilize or improve disability in most patients. However, the effect from ALZ on cognition seemed limited since SDMT score decreased at follow-up. Although, we observed a normalization of NfL levels at follow-up, there were no change of GFAP concentrations, suggesting that ALZ reduce axonal damage but has no or only limited influence on astrogliosis. We also confirmed that NfL at baseline may predict disease severity in RRMS. Except for low age (≤30 years) we found no other baseline demographic or clinical factors of prognostic value. Our study confirms that ALZ, an immune reconstitution therapy, after few initial immunosuppressive courses has long-term effects on most clinically relevant outcomes in RRMS. The possible influence from ALZ on progressive axonal and functional loss of neurons and astrogliosis warrants further investigations.

## Data availability statement

The raw data supporting the conclusions of this article will be made available by the authors, without undue reservation.

## Ethics statement

The studies involving humans were approved by the Regional Ethics Review Board in Gothenburg, Sweden (Reference number 460-13). The studies were conducted in accordance with the local legislation and institutional requirements. The participants provided their written informed consent to participate in this study. The study conformed with the Code of Ethics of the World Medical Association (Declaration of Helsinki) (52).

## Author contributions

SS: Data curation, Formal analysis, Investigation, Methodology, Visualization, Writing – original draft. LN: Investigation, Methodology, Writing – review & editing. AN: Data curation, Methodology, Writing – review & editing. MA: Investigation, Writing – review & editing. CM: Conceptualization, Methodology, Writing – review & editing. HZ: Conceptualization, Methodology, Writing – review & editing. JL: Conceptualization, Funding acquisition, Methodology, Supervision, Writing – original draft, Writing – review & editing.

## Figures and Tables

**Table:** 

ALZ	alemtuzumab
ARR	annualized relapse rate
CDI	confirmed disability improvement
CDW	confirmed disability worsening
CI	confidence interval
CNS	central nervous system
CSF	cerebrospinal fluid
CTCAE	common terminology criteria for adverse events
DMT	disease-modifying treatment
EDA-3	evidence of disease activity-3
EDSS	expanded disability status scale
fLLoQ	functional lower limit of quantification
GFAP	glial fibrillary acidic protein
IQR	interquartile range
JC virus	John Cunningham virus
MRI	magnetic resonance imaging
MS	multiple sclerosis
NEDA-3	no evidence of disease activity-3
NfL	neurofilament light
PIRA	progression independent of relapses
RRMS	relapsing–remitting multiple sclerosis
SCs	symptomatic controls
SD	standard deviation
SDMT	symbol digit modality test
Simoa	single molecule array
QC	quality control
