# Supplementary material for: A five-year observational prospective mono-center study of the efficacy of alemtuzumab in a real-world cohort of patients with multiple sclerosis
Source: Front Neurol. 2023 Sep 21;14:1265354. doi: 10.3389/fneur.2023.1265354 (PMC10551138; doi:10.3389/fneur.2023.1265354)
Supplement: Supplementary file 1 [file Data_Sheet_1.PDF]

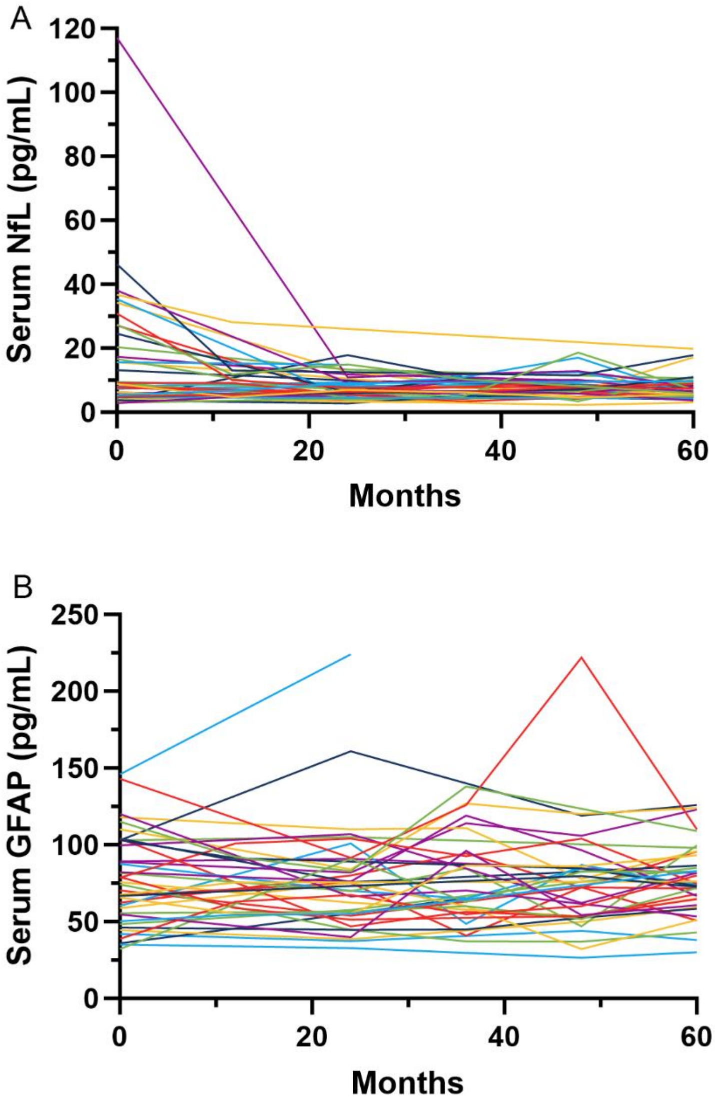

Supplementary Figure 1 Individual serum NfL and GFAP levels. The individual concentration of (**A)** serum NfL, and (**B)** serum GFAP, at baseline, and during follow-up, for each patient who had a baseline value (n = 41). Each individual line represents an individual. NfL, neurofilament light; GFAP, glial fibrillary acidic protein.
